# Bacterial community associated with worker honeybees (*Apis mellifera*) affected by European foulbrood

**DOI:** 10.7717/peerj.3816

**Published:** 2017-09-25

**Authors:** Tomas Erban, Ondrej Ledvinka, Martin Kamler, Bronislava Hortova, Marta Nesvorna, Jan Tyl, Dalibor Titera, Martin Markovic, Jan Hubert

**Affiliations:** 1 Crop Research Institute, Prague, Czechia; 2 Czech Hydrometeorological Institute, Prague, Czechia; 3 Bee Research Institute at Dol, Libcice nad Vltavou, Czechia; 4 Department of Zoology and Fisheries/Faculty of Agrobiology Food and Natural Resources, Czech University of Life Sciences, Prague, Czechia

**Keywords:** *Melissococcus plutonius*, Pathogen detection, *Snodgrassella alvi*, *Lactobacillus*, *Fructobacillus fructosus*, *Bartonella apis*, *Frischella perrara*, Microbiome, *Enterococcus faecalis*, *Gilliamella apicola*

## Abstract

**Background:**

*Melissococcus plutonius* is an entomopathogenic bacterium that causes European foulbrood (EFB), a honeybee (*Apis mellifera* L.) disease that necessitates quarantine in some countries. In Czechia, positive evidence of EFB was absent for almost 40 years, until an outbreak in the Krkonose Mountains National Park in 2015. This occurrence of EFB gave us the opportunity to study the epizootiology of EFB by focusing on the microbiome of honeybee workers, which act as vectors of honeybee diseases within and between colonies.

**Methods:**

The study included worker bees collected from brood combs of colonies (i) with no signs of EFB (EFB0), (ii) without clinical symptoms but located at an apiary showing clinical signs of EFB (EFB1), and (iii) with clinical symptoms of EFB (EFB2). In total, 49 samples from 27 honeybee colonies were included in the dataset evaluated in this study. Each biological sample consisted of 10 surface-sterilized worker bees processed for DNA extraction. All subjects were analyzed using conventional PCR and by metabarcoding analysis based on the 16S rRNA gene V1–V3 region, as performed through Illumina MiSeq amplicon sequencing.

**Results:**

The bees from EFB2 colonies with clinical symptoms exhibited a 75-fold-higher incidence of *M. plutonius* than those from EFB1 asymptomatic colonies. *Melissococcus plutonius* was identified in all EFB1 colonies as well as in some of the control colonies. The proportions of *Fructobacillus fructosus*, *Lactobacillus kunkeei*, *Gilliamella apicola*, *Frischella perrara*, and *Bifidobacterium coryneforme* were higher in EFB2 than in EFB1, whereas *Lactobacillus mellis* was significantly higher in EFB2 than in EFB0. *Snodgrassella alvi* and *L. melliventris*, *L. helsingborgensis* and, *L. kullabergensis* exhibited higher proportion in EFB1 than in EFB2 and EFB0. The occurrence of *Bartonella apis* and *Commensalibacter intestini* were higher in EFB0 than in EFB2 and EFB1. *Enterococcus faecalis* incidence was highest in EFB2.

**Conclusions:**

High-throughput Illumina sequencing permitted a semi-quantitative analysis of the presence of *M. plutonius* within the honeybee worker microbiome. The results of this study indicate that worker bees from EFB-diseased colonies are capable of transmitting *M. plutonius* due to the greatly increased incidence of the pathogen. The presence of *M. plutonius* sequences in control colonies supports the hypothesis that this pathogen exists in an enzootic state. The bacterial groups synergic to both the colonies with clinical signs of EFB and the EFB-asymptomatic colonies could be candidates for probiotics. This study confirms that *E. faecalis* is a secondary invader to *M. plutonius*; however, other putative secondary invaders were not identified in this study.

## Introduction

European foulbrood (EFB) is caused by the Gram-positive lanceolate coccus *Melissococcus plutonius* (Bailey & Collins, 1982; Truper & De’ Clari, 1998), and classification of the causative agent of EFB was difficult. In the earliest studies of this disease, the causative agent was considered *Streptococcus apis* (Burri) or *Bacillus pluton* (White, 1912), and later, between 1957 and 1982, the agent was referred to as *S. pluton* (Bailey, 1957b). EFB is one of the most important diseases of the European honeybee, *Apis mellifera* L. (Bailey, 1956; Bailey, 1957a; Bailey, 1960; Bailey, 1983; Bailey, 1985; Forsgren, 2010; White, 1912). EFB mainly causes problems in spring and is primarily associated with weak colonies and those with low food reserves (Bailey, 1960; Poltev, 1950). This emerging honeybee brood disease is listed in the Terrestrial Animal Health Code of The World Organization for Animal Health (OIE) (OIE, 2016b). Although the spread of EFB is global and the clinical signs are similar to those of American foulbrood (AFB), EFB is not notifiable in all countries (Forsgren et al., 2013). Because EFB can be confused with AFB or other brood abnormalities (Alippi, 1991; Forsgren et al., 2013) and because *M. plutonius* is difficult to detect in brood frames showing significant disease symptoms (Forsgren et al., 2005), reliable early detection of diseased colonies is critical for minimizing the risk of disease development.

EFB is cosmopolitan in areas where honeybees are kept; however, in the past several years, the incidence of EFB has increased in some European countries. In particular, it has continuously increased in Switzerland since 1997 (Belloy et al., 2007; Roetschi et al., 2008). In the UK, EFB has become the most common brood disease (Budge et al., 2011; Wilkins, Brown & Cuthbertson, 2007), and a regional outbreak occurred in Norway in 2010 after a 30-year absence of EFB (Dahle, Sorum & Weideman, 2011). In addition, EFB has historically occurred in Czechia (which was part of Czechoslovakia at that time). In June 1975, EFB was found in Kralupy nad Vltavou in Central Bohemia (F Kamler, pers. obs., 1975). EFB was also found in Czechia recently (in 2015), and additional signs of the disease emerged in 2016 in the Krkonose Mountains National Park in Eastern Bohemia (KVSH, 2015).

The first significant study of EFB etiology was conducted in 1912 by White, who noted that sick larvae were more transparent or possessed a yellowish tint compared with the color of healthy larvae of the same age (White, 1912). Indicators of the disease may also be present in a subset of larvae due to the partial removal of larvae by adult bees (White, 1912). Bailey suggested that workers remove infected larvae because they require more food due to the disease (Bailey, 1960), and an adequate food supply can support survival of the larvae, leading to pupation (Bailey, 1983). The adult worker bees removing infected larvae act as vectors of *M. plutonius* cells via food transmission to healthy broods within and between colonies; this ability to carry *M. plutonius* was demonstrated by McKee et al. (2003). The first step in EFB infection is asymptomatic colonization of the larval gut and growth in the midgut after food transmission by nurse bees (Bailey, 1959; Bailey, 1960; Tarr, 1938). The infection localizes to the surface of the peritrophic membrane of the larval gut; however, indications of the diffusion of *M. plutonius*-derived substances into larval tissues have been observed (Takamatsu, Sato & Yoshiyama, 2016). Secondary infections can have a supplementary pathogenic effect on *M. plutonius* in diseased larvae (Bailey, 1956; Bailey, 1957a; Bailey, 1963; Tarr, 1938; White, 1912), thereby influencing larval survival (Bailey, 1983). However, the role in disease development that is played by secondary invaders, such as *Enterococcus faecalis*, *Achromobacter eurydice* (previously *Bacterium eurydice*), *Brevibacillus laterosporus*, *Paenibacillus alvei*, and *Paenibacillus dendritiformis*, which are likely associated with signs of EFB in larvae (Alippi, 1991; Bailey, 1956; Bailey, 1963; Bailey & Ball, 1991; Djordjevic et al., 1998; Gaggia et al., 2015), is unclear (Erler et al., in press). It is curious that White already in 1912 mentioned the co-occurrence of *A. eurydice* in the disease stage, which was found in low numbers compared with *M. plutonius* (White, 1912). According to Bailey, EFB is caused by a combination of *M. plutonius* and *A. eurydice* (Bailey, 1957a), whereas other works highlight *E. faecalis* (Bailey, 1963; Forsgren, 2010; Gaggia et al., 2015; OIE, 2008). It should be noted that the recent literature review by Erler et al. (in press), suggested the confusion of *A. eurydice* with the fructophilic lactic bacterium *Lactobacillus kunkeei* in relation to EFB.

Some infected larvae can survive and deposit bacteria on comb cells via their feces, and *M. plutonius* survives in these deposits (Bailey, 1959). Although the duration of EFB disease development can vary, larvae usually die due to bacteremia at between four and five days of age and sometimes at an older age, after sealing (Forsgren, 2010). Some infected individuals may even survive to the pupal stage, although their weight is reduced by the infection (Bailey, 1960). Recent research indicated that the epidemiology of EFB is affected by the virulence of the *M. plutonius* strain, presumably resulting in differential disease development between countries (Arai et al., 2012; Budge et al., 2014; Nakamura et al., 2016; Takamatsu, Sato & Yoshiyama, 2016). Lower *M. plutonius* virulence is likely the reason that some individual larvae are capable of pupation (Nakamura et al., 2016).

The methods used to detect EFB were reviewed by Forsgren et al. (2013) and are included in the COLOSS BeeBook (Dietemann, Ellis & Neumann, 2013) and the OIE Terrestrial Manual (OIE, 2016a). Compared with the causative agent of AFB, *Paenibacillus larvae*, which forms spores, *M. plutonius* is non-sporulating and is thus more difficult to identify using cultivation techniques because fewer than 0.2% of cells are detectable (Djordjevic et al., 1998; Hornitzky & Smith, 1999). White (1912) noted that the most favorable period for making a diagnosis based on gross examination alone is three days before a larva would ordinarily be capped (White, 1912). According to the OIE, freshly dead larvae are preferred for diagnostic analysis (OIE, 2016a). Microscopy techniques, such as the observation of carbol fuchsin smears of larvae and honey (Hornitzky & Wilson, 1989) and scanning electron microscopy (Alippi, 1991), are the optical detection methods employed for diagnosis. The biochemical methods used to detect *M. plutonius* include immunochemical methods, such as ELISA and LFIA (Pinnock & Featherstone, 1984; Tomkies et al., 2009), immunohistochemical localization (Takamatsu, Sato & Yoshiyama, 2016), and PCR-based approaches, including conventional PCR (Govan et al., 1998), hemi-nested PCR (Belloy et al., 2007; Djordjevic et al., 1998; Forsgren et al., 2005; McKee et al., 2003) and qPCR (Budge et al., 2010; Roetschi et al., 2008). Moreover, multilocus sequence typing (MLST) has been used to classify different *M. plutonius* isolates (Haynes et al., 2013; Nakamura et al., 2016).

Based on a qPCR analysis of colonies exhibiting clinical signs of EFB, bees collected from brood nests were found to harbor an approximately 20-fold higher *M. plutonius* load than bees from flight entrances (Roetschi et al., 2008). However, this finding obtained by the analysis of 100 bees per sample has not been repeated (Forsgren et al., 2013). Through hemi-nested PCR, Belloy et al. (2007) identified honeybees carrying *M. plutonius* in more than 90% of colonies without EFB symptoms within EFB symptomatic apiaries. Moreover, bees carrying *M. plutonius* were found in approximately 30% of colonies in apiaries without EFB symptoms located near apiaries with clinical symptoms of EFB (Belloy et al., 2007). The number of *M. plutonius* cells in adult bees varies, but bees from asymptomatic colonies in EFB-diseased apiaries are at higher risk of disease development (Budge et al., 2010). A metatranscriptomic approach has been used to analyze the microbial community associated with honeybees, revealing the suitability of this methodology for detecting *M. plutonius* (Tozkar et al., 2015). However, this approach has not been tested in honeybee worker samples from confirmed EFB outbreaks, which is a situation different from that described in Tozkar et al. (2015), who indicated that the different distribution of *M. plutonius* among regions could be explained as a factor of the environmental conditions or different bee immunity.

In this study, we investigated EFB in the context of a disease outbreak in the Krkonose Mountains National Park in Czechia, representing the first case to be identified after 40 years without any verified incidence of EFB in Czechia (Kamler et al., 2016). We followed a previously published experimental design (Belloy et al., 2007; Roetschi et al., 2008) to analyze honeybees obtained from both symptomatic and asymptomatic colonies in EFB-diseased apiaries. For comparison, we also performed an analysis of worker honeybees from control colonies located far from the occurrence of the outbreak. The worker microbiome was described based on a high-throughput sequencing (HTS) analysis of the V1-V3 region of the 16S rRNA gene using the Illumina MiSeq platform. In addition to determining the relative numbers of *M. plutonius* sequences that were correlated with different sample types, we identified the effects of the presence of *M. plutonius* on the symbiotic bacterial community of the honeybee gut. Thus, the influence of EFB on the host microbiome was indicated.

**Figure 1:**
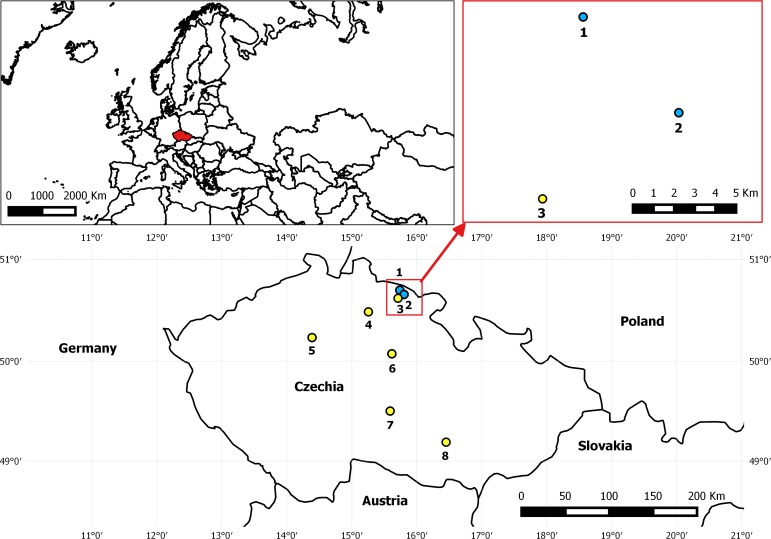
Map depicting the location of EFB outbreak in Czechia in August 2015 and control apiaries included in analyses. The two apiaries indicated with blue dots as No. 1 Pec pod Snezkou (four EFB2 colonies) and No. 2 Horni Marsov (four EFB2 and two EFB1 colonies) presented the first two confirmed cases of EFB in 40 years. Apiary No. 3 Cerny Dul-Cista was sampled as a control (EFB0) in this area, and other EFB0 control samples originated from apiaries Nos. 4–8 (4—Strelec, 5—Postrizin, 6—Prelovice, 7—Stoky-Skrivanek, 8—Kyvalka).

## Materials & Methods

### Apiaries and sampling

The samples of managed European honeybees (*Apis mellifera* ssp. *carnica*) included in the analysis originated from two EFB-diseased apiaries and from control colonies geographically isolated from the outbreak zone (Fig. 1 and Table S1). In total, 49 HTS-analyzed samples from 27 honeybee colonies were included in the dataset evaluated in this study (Table S1). All of the colonies from EFB-diseased apiaries were analyzed using biological triplicates of collected worker bees. The honeybee samples from EFB-diseased apiaries located in the outbreak region in the Krkonose Mountains National Park in Eastern Bohemia, Czechia, comprised four colonies from an apiary in Pec pod Snezkou with only visible clinical symptoms, whereas the second EFB-diseased apiary in Horni Marsov included four colonies with clinical symptoms of EFB and two colonies without visible EFB manifestation (Fig. 1 and Table S1). The State Veterinary Administration of the Czech Republic declared the two EFB apiaries to be the epicenter of the outbreak zone, and plans to move any of the colonies to a protective zone (a 5-km radius from each disease outbreak) were abandoned according to Czech legislation. In addition to the samples from the two apiaries with clinical symptoms of EFB, we collected samples from control colonies and included some samples already analyzed and previously published, i.e., Accessions: PRJNA304944 (Hubert et al., 2016) and PRJNA326764 (Hubert et al., 2017); for details, see Table S1. The experimental sampling design and the procedure for the sampling of bees from colonies used in the present study were similar to those employed in a previous study from Switzerland (Belloy et al., 2007), in that the distribution of *M. plutonius* was evaluated among bees originating from apiaries and colonies with and without symptoms of EFB. Bees were sampled from the brood combs because such samples are considered more suitable for EFB detection than those from the hive entrance (Roetschi et al., 2008). The honeybees were shaken off the brood combs into plastic bags, and the bags were placed in a box with dry ice for transport and then stored at −80 °C.

For our analyses, we coded the samples by the EFB factor in the following manner: (i) EFB0—control bees collected outside the EFB zone with no signs of EFB, (ii) EFB1—bees from an EFB apiary but from colonies without clinical symptoms (asymptomatic), and (iii) EFB2—bees from colonies with clinical symptoms of EFB.

### DNA extraction from honeybees

Each biological sample used for DNA extraction included 10 hive worker bees, and the analyses were performed using biological triplicates of each colony from the EFB outbreak apiaries. To process the samples, we followed a previously described procedure (Hubert et al., 2016; Hubert et al., 2017). Prior to DNA extraction, the samples were surface-sterilized by washing with bleach (Savo, Unilever, Prague, Czechia) (once for 30 s), ethanol (once for 60 s), and phosphate-buffered saline (PBS)-Tween 20 (Cat. No. P2287, Sigma-Aldrich, St. Louis, MO, USA) (twice for 120 s). The bees were then transferred to sterile polypropylene vials (Cat No. 3205, BioSpec Products, Bartlesville, OK, USA). Each vial contained 0.6 g of a mixture of glass and garnet beads 0.1–1 mm in diameter (BioSpec, Cat. Nos. 11079101, 11079103gar, 11079105, and 11079110gar; 1∕1∕1∕1 wt/wt/wt/wt). Next, 2 mL of PBS-Tween 20 and 4 mL of phenol/chloroform/isoamyl alcohol (Roti-Phenol^®^, Cat No. A156.2, Carl Roth, Karlsruhe, Germany) were added, and the samples were homogenized for 2 min using a Mini-Beadbeater-16 (BioSpec). The homogenates were next transferred to sterile 15-mL centrifuge tubes (Orange Scientific, Braine-l’Alleud, Belgium) and centrifuged (4,508 × g for 5 min). The supernatants were mixed with 6 mL of sterilized ddH_2_O containing 100 µL of Tween 20 and then centrifuged at 4,508 × g for 5 min. The upper aqueous phase was then extracted twice with chloroform/isopropanol (24:1 ratio) and centrifuged. The upper aqueous phase was subsequently transferred to a 1.5-mL reaction tube and precipitated with 100 µL of 3 M ammonium acetate (Cat No. S7899, Sigma-Aldrich, St. Louis, MO, USA) and 500 µL of isopropanol. For precipitation, the mixture was incubated at −40 °C for 15 min. The tubes were subsequently centrifuged (13,845 × g, 15 min), and the pellets were washed twice with 70% ethanol. The pellets were then dried under a vacuum (60 min) to remove the remaining ethanol and placed at a controlled temperature of 56 °C. After 200 µL of ddH_2_O was added, the samples were incubated for 15 min, and the pellets were dissolved by pipetting. The resultant solution was vortexed repeatedly. Finally, the DNA was cleaned using a GeneClean^®^ Turbo kit (Cat No. 1102–600, MP Biomedicals, Santa Ana, CA, USA) and stored at −40 °C until use.

### Amplification, sequencing and data processing

After DNA purification, PCR amplification using the universal primers 27F and 1492R (Lane, 1991) and using specific EFB primers (Govan et al., 1998; Lane, 1991) was performed to detect the presence of bacterial DNA and to demonstrate the presence of *M. plutonius*, respectively. If an amplicon was not obtained, the sample was replaced with a new one that showed positive amplification with the universal primers (Cariveau et al., 2014a; Cariveau et al., 2014b). The DNA samples were sent to MR DNA (http://mrdnalab.com, Shallowater, TX, USA) for sequencing of the V1-V3 region of the 16S rRNA gene using the Illumina MiSeq platform according to the manufacturer’s guidelines. The universal primers 27Fmod and 519Rmod, with barcodes, were employed, and the products were sequenced on the Illumina MiSeq platform (Chiodini et al., 2015) by MR DNA (http://www.mrdnalab.com/amplicon-sequencing-(btefap%C2%AE).html). The read length was 300 bp, and both forward and reverse reads were obtained. The sequences were processed as previously described (Erban et al., 2017b; Hubert et al., 2016) using MOTHUR v.1.36.1 software (Schloss et al., 2009) according to the standard MiSeq operating procedure (Kozich et al., 2013) and using UPARSE (Edgar, 2013). Chimeras were identified using the SILVA reference database (Quast et al., 2013) and UCHIME (Edgar et al., 2011). OTUs were identified according to the Ribosomal Database Project (http://rdp.cme.msu.edu) (Cole et al., 2014) employing training set No. 15 available for UPARSE (https://www.drive5.com/usearch/manual/sintax_downloads.html). Representative sequences were then processed using the blastn program on the NCBI platform (https://blast.ncbi.nlm.nih.gov/) (Altschul et al., 1997). The best search hits were selected based on the highest bit score. The obtained sequences were deposited in GenBank (Benson et al., 2013) under Sequence Read Archive (SRA) No. SRP093440; Accession No. PRJNA352995 (the microbiome of *A. mellifera* associated with European foulbrood), and a list of the samples is presented in Table S1. The taxonomic features of the samples were visualized via Krona projection (Ondov, Bergman & Phillippy, 2011). The sequence numbers among the samples varied between 17,759 (PO1) and 86,310 (PR4B). The data were standardized by subsampling to a subsample of 17,759 sequences (the lowest number of sequences per sample) in MOTHUR. The subsequent analyses were performed using these standardized data.

### Data analysis

The effect of EFB factor on α-diversity (inverse Simpson index and number of OTUs) was analyzed. The indexes were calculated using MOTHUR based on standardized data and then compared through a nonparametric Kruskal–Wallis test and the Dunn post-hoc procedure using XLSTAT software (http://www.xlstat.com/en/, Addinsoft, New York, NY, USA). Homogeneity of molecular variance (HOMOVA) (Schloss, 2008; Stewart Jr & Excoffier, 1996) analysis was used to test whether the genetic diversity within the EFB levels was homogeneous. Prior to the assessment of β-diversity (comparison of the samples), the OTU subsample data were transformed into Bray–Curtis and Jaccard dissimilarity matrixes. The two measures were investigated because the Bray–Curtis index, although quite popular in this type of study (Engel et al., 2013), is semimetric (Legendre & Legendre, 2012) and includes intrinsic standardizations that may affect patterns of relative dispersion in ways that cannot be easily interpreted by reference to the original data (Anderson, Ellingsen & McArdle, 2006). Therefore, by also utilizing the metric Jaccard index, we wanted to explore whether there were any important differences. Based on these matrixes, an analysis of similarities (ANOSIM) using PAST 3.14 (Hammer, Harper & Ryan, 2001) was then performed with 100,000 permutations. Visualization was accomplished through non-metric multidimensional scaling (NMDS) in PAST. Additionally, a more thorough evaluation based on distance-based redundancy analysis (db-RDA) was performed using the R package “vegan” (Oksanen et al., 2016). In particular, a partial version of db-RDA was performed, in which the influences of geographic coordinates and the time of bee collection (in terms of Julian days) were suppressed. The environmental variables included geographical position, sampling time expressed in Julian days, EFB factor and the results of PCR performed with EFB primers (conventional PCR confirmation). Moreover, a logarithmic transformation (LOG2), as recommended previously (Anderson, Ellingsen & McArdle, 2006), was applied only to the column representing OTU4 (*M. plutonius*) before transforming it to the Bray–Curtis distance, and another partial RDA model was constructed using this column together with all possible explanatory variables. In both cases (the RDA with all OTUs and the RDA with OTU4 alone), the significance of the explanatory variables was also studied by performing a forward selection procedure using the R package “packfor” (Dray, Legendre & Blanchet, 2013). The redundancy of the explanatory variables was controlled using variance inflation factors (VIFs) (Kutner et al., 2005). An attempt was made to identify the best partial RDA model that would best explain the variance in OTUs, in terms of the smallest P-value. The relative OTU abundance in the samples was tested using METASTATS in MOTHUR with 100,000 permutations and with random forest algorithms employing 1,500 trees. Using the relative OTU abundance data and logarithmically transformed OTU data obtained from the subsample, heatmaps were constructed to determine whether the OTUs clustered across sites. The heatmaps were generated with the R package “gplots” (Warnes et al., 2016).

### Map

The map was made using QGIS (QGIS Development Team, 2009) v2.8-Wien and the coordinate system WGS84/World Mercator 3395.

## Results

### Microbiome analyses

The rarefaction curves for EFB0, EFB1 and EFB2 shown in Figs. S1–S3 depict the α-diversity of the three types of samples. The proportions of the worker bee microbiome were visualized using Krona projections for different situations according to the EFB factor (Fig. S4). *Melissococcus plutonius* was not recognizable within the microbiome proportions in the Krona projections of EFB0 (Fig. S4A) and EFB1 (Fig. S4B), but this species accounted for approximately 3% of the microbiome in EFB2 (Fig. S4C). The comparison of the number of sequences *M. plutonius* sequences represents Fig. S5. Interestingly, the microbiome of EFB2 contained 4% *E. faecalis* and 2% *F. fructosus* (Fig. S4C). In addition, small numbers of *P. larvae* were detected in some samples (see the raw dataset in Table S2).

**Figure 2:**
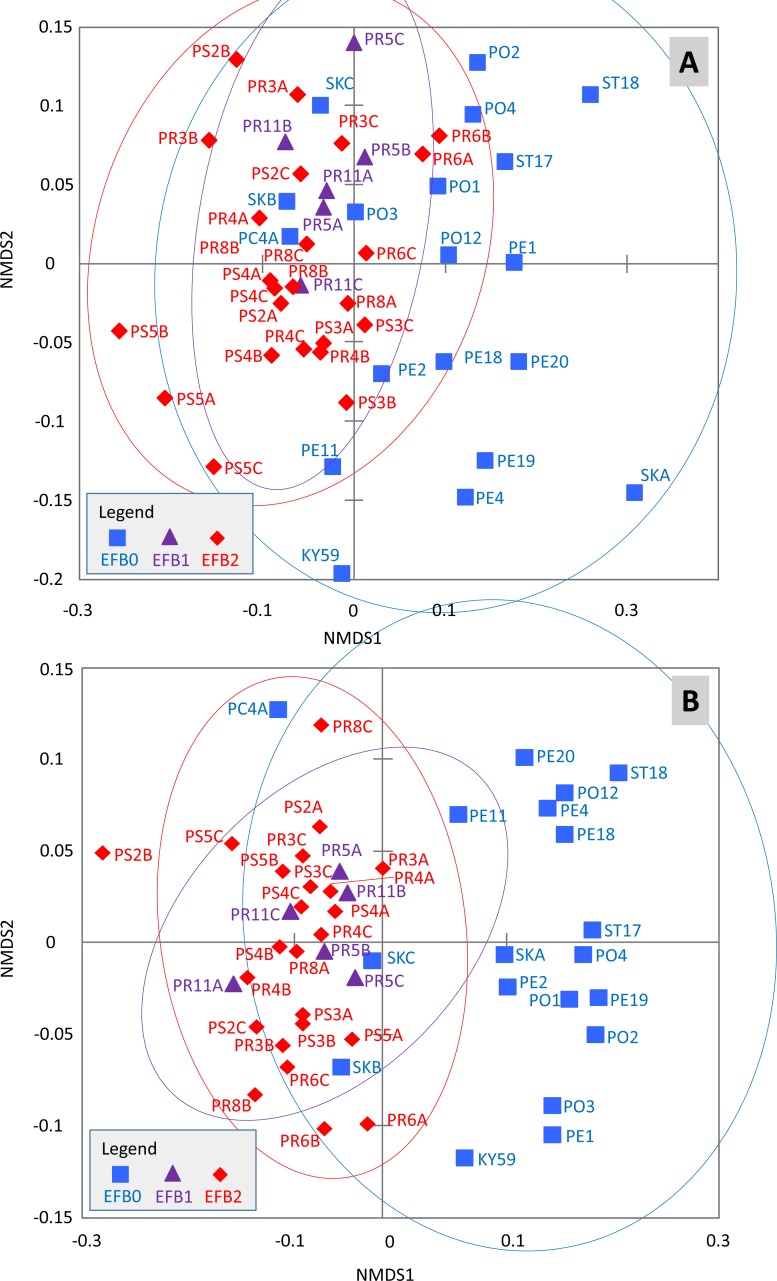
Distribution of the samples determined through non-metric multidimensional scaling using Bray–Curtis (A) and Jaccard (B) matrixes. The 95% confidence eclipses are included. The detailed description of the samples is provided in Tables S1 and S2 (raw dataset). Legend: (i) EFB0—control bees from outside of the EFB zone without signs of EFB; (ii) EFB1—bees from an EFB apiary, but from colonies without clinical symptoms; and (iii) EFB2—bees from colonies with clinical symptoms of EFB.

The effects of the coded EFB factors on the OTU distribution in the worker bee microbiome were tested using the Bray–Curtis dissimilarity measure. The HOMOVA procedure showed that the assumption of variance homogeneity across different EFB levels could be accepted (BValue = 2.747, *P* = 0.087). ANOSIM indicated differences in the microbiome based on the coded EFB factor (*R* = 0.22, *P* < 0.001 and *R* = 0.486, *P* < 0.001 for the Bray–Curtis and Jaccard matrixes, respectively). The inverse Simpson diversity index was not influenced by the coded EFB factor (Kruskal–Wallis test; *K* = 3.183, *P* = 0.208). Pairwise comparisons after Bonferroni correction (*P* < 0.05) indicated differences in the bee microbiome between colonies with clinical symptoms (EFB2) and control colonies (EFB0) (*P* < 0.001). No differences in either the Bray–Curtis or Jaccard matrix were observed between colonies without clinical symptoms (EFB1) and control colonies (EFB0) (*P* = 1). When comparing colonies with clinical symptoms (EFB2) and those without clinical symptoms (EFB1), the differences were not significant (*P* = 0.894) for the Bray–Curtis matrix but were significant (*P* < 0.001) for the Jaccard matrix. The distribution of samples was visualized using non-metric multidimensional scaling functions (Fig. 2AB), which confirmed the results of pairwise tests.

In the RDAs, one of the VIFs (Table S3) slightly crossed the limit of 10; specifically, the VIF connected to the third level of the EFB factor (EFB2) reached a value of 10.6. Because some authors report that strong multicollinearity is indicated by VIFs larger than 20 (Borcard, Gillet & Legendre, 2011), all of the explanatory variables were included in the RDA models (i.e., EFB0-2 plus PCR detection). The Julian days, representing the sample collection times, and the geographic coordinates in terms of latitude and longitude conditioned the influence of the EFB factor and PCR detection for all of the models (Table 1). These conditionings were demonstrated to be good choices because longitude had a significant negative effect on the matrix of OTU abundance. Specifically, the canonical coefficients measuring the relationship between longitude and the six resulting canonical axes were as follows (from first to sixth): −0.46, −0.13, −0.11, −0.14, 0.04 and −0.13. However, this hypothesis must be tested based with a larger sample representing a much wider territory. Moreover, only the first axis of the RDA was significant (*P* < 0.01) when all explanatory variables were added to the model with all OTUs. The situation was similar when *M. plutonius* (OTU4) (or its logarithm) was studied as the only dependent variable. However, taking into account the partial RDA counterparts, none of the models (either with all OTUs or with *M. plutonius* OTU4 alone) were significant at the 0.05 level (Table 1). Furthermore, none of the terms added as explanatory variables (excluding those acting as conditions) appeared to be significant, and the newly obtained axes were also not significant.

**Table 1:** Results of permutation tests applied to explanatory variables and the axes obtained from RDA models for the whole OTU matrix and for *Melissococcus plutonius* (OTU4) alone (1,000 replications used). Initial models contained all possible explanatory variables. Partial models were constructed only for EFB and EFB/PCR variables, while factoring out the coordinates and Julian days. The number of RDA axes generally depends on the number of variables (or their levels) and, therefore, there were no possible results for some instances (indicated by “—”).

	Initial				Partial			
	Variable	*P*-value	Axis	*P*-value	Variable	*P*-value	Axis	*P*-value
RDA with all OTUs	EFB	0.678	1	**0.004**	EFB	0.711	1	0.301
	EFB/PCR	0.652	2	0.376	EFB/PCR	0.684	2	0.744
	Julian days	0.377	3	0.444	–	–	3	0.965
	Latitude	0.925	4	0.792	–	–	–	–
	Longitude	**0.005**	5	0.973	–	–	–	–
	–	–	6	0.994	–	–	–	–
RDA with OTU4 only	EFB	0.203	1	**0.002**	EFB	0.186	1	0.335
	EFB/PCR	0.958	–	–	EFB/PCR	0.928	–	–
	Julian days	0.314	–	–	–	–	–	–
	Latitude	0.768	–	–	–	–	–	–
	Longitude	**0.002**	–	–	–	–	–	–

A community-level analysis indicated differences in the relative numbers of OTUs according to the EFB factor, and random forest tree algorithms revealed OTUs that significantly contributed to differences in the microbiome (error rate = 0.12) (Table 2). The METASTATS analysis (Table 2) showed significant differences in the relative proportions of some bacteria in the microbiome: the proportions of *F. fructosus* (OTU5), *Lactobacillus kunkeei* (OTU9) and *Gilliamella apicola* (OTU3) were significantly higher in EFB2 than in EFB1 and EFB0; the proportions of two other OTUs, related to *G. apicola* (OTU16 and OUT55) and *Bifidobacterium coryneforme* (OTU17), were higher in EFB2 than in EFB1; the proportion of *Lactobacillus mellis* (OTU8) was significantly higher in EFB2 than in EFB0, whereas that of *Commensalibacter intestini* (OTU15) was higher in EFB0 than in EFB2; the proportions of *Snodgrassella alvi* (OTU6) and *Lactobacillus melliventris* (OTU25) were higher in EFB1 compared with EFB2 and EFB0 and in EFB2 compared with EFB0; the proportion of *Frischella perrara* (OTU18) was highest in EFB0 and was higher in EFB2 than in EFB1; the proportions of *Bartonella apis* (OTU2) were similar in EFB2 and EFB1 but higher in EFB0; the proportion of *E. faecalis* (OTU10) was highest in EFB2, and that in EFB1 was significantly lower than in EFB0; and the proportions of *Lactobacillus helsingborgensis* (OTU7) and *L. kullabergensis* (OTU12) were higher in EFB1 than in EFB2 and EFB0. The changes in the proportions of these OTUs are also visible in the Krona projections (Fig. S4).

**Table 2:** Relative proportions of selected OTUs in the honeybee microbiome. The following codes were used for the different sample types: (i) EFB0—control bees from outside of the EFB zone without signs of EFB; (ii) EFB1—bees from an EFB apiary, but from colonies without clinical symptoms; and (iii) EFB2—bees from colonies with clinical symptoms of EFB. The sequences were analyzed using METASTATS, and *P*-values are presented. The OTUs are described according to the closest match in GenBank, and the numbers in brackets indicate the % identity. *P*-values in bold indicate a statistically significant effect of the EFB factor, with a *p* value of less than 0.05.

OTU_97_	GenBank identification	aOTU	Mean decrease accuracy	EFB factor								
				EFB2		EFB1		EFB0		*P*-values		
				Mean	%stderr	Mean	%stderr	Mean	%stderr	EFB2 EFB1	EFB2 EFB0	EFB1 EFB0
OTU4	*Melissococcus plutonius* (99)	13,112	1.361	0.03055	23.1	0.00046	19.3	0.00012	54.8	**0.001**	**0.000**	**0.011**
OTU5	*Fructobacillus fructosus* (99)	9,845	0.737	0.02272	36.0	0.00133	49.1	0.00006	50.8	**0.028**	**0.000**	0.079
OTU109	*Lactobacillus kimbladii* (99)	2,668	0.313	0.00296	7.1	0.00487	20.6	0.00264	21.4	0.096	0.598	0.079
OTU9	*Lactobacillus kunkeei* (99)	5,679	0.275	0.01260	39.6	0.00045	21.8	0.00077	82.6	**0.040**	**0.002**	0.714
OTU8	*Lactobacillus mellis* (99)	44,528	0.273	0.06059	8.7	0.05986	16.5	0.03653	19.3	0.962	**0.010**	0.083
OTU6	*Snodgrassella alvi* (99)	101,925	0.269	0.12245	8.2	0.22007	11.4	0.07790	12.8	**0.003**	**0.003**	**0.000**
OTU7	*Lactobacillus helsingborgensis* (99)	61,676	0.245	0.07324	12.4	0.10213	5.9	0.05802	10.8	**0.025**	0.183	**0.000**
OTU12	*Lactobacillus kullabergensis* (99)	26,246	0.243	0.02732	9.0	0.04641	7.6	0.02862	12.8	**0.000**	0.772	**0.004**
OTU28	unident. Gammaproteobacteria	2,147	0.235	0.00031	73.9	0.00008	47.6	0.00595	60.9	0.474	0.058	0.143
OTU16	*Gilliamella apicola* (97)	7,951	0.225	0.00209	29.8	0.00033	47.9	0.02082	53.4	**0.020**	0.088	0.097
OTU3	*Gilliamella apicola* (99)	203,595	0.222	0.26670	6.3	0.18932	14.2	0.20672	10.6	**0.040**	**0.036**	0.710
OTU15	*Commensalibacter intestini* (95)	9,021	0.222	0.00318	25.7	0.00313	37.6	0.02174	65.8	0.975	**0.011**	0.241
OTU25	*Lactobacillus melliventris* (97)	10,699	0.205	0.01301	12.4	0.02103	12.7	0.00863	13.4	**0.031**	**0.024**	**0.001**
OTU18	*Frischella perrara* (99)	26,368	0.201	0.02100	12.8	0.01231	14.1	0.04773	19.9	**0.021**	**0.005**	**0.002**
OTU33	*Bifidobacterium asteroides* (98)	18,563	0.200	0.02090	12.4	0.02553	14.9	0.02056	25.5	0.391	0.956	0.564
OTU13	*Lactobacillus mellifer* (99)	8,712	0.198	0.00849	11.2	0.00991	18.8	0.01197	16.4	0.634	0.122	0.569
OTU2	*Bartonella apis* (99)	77,679	0.197	0.05323	34.1	0.05017	46.4	0.14713	17.3	0.942	**0.005**	**0.015**
OTU14	*Bifidobacterium asteroides* (99)	22,375	0.193	0.02504	11.0	0.04562	32.0	0.02027	21.1	0.219	0.354	0.133
OTU10	*Enterococcus faecalis* (99)	16,167	0.193	0.03665	54.9	0.00029	19.6	0.00153	13.4	0.107	0.240	**0.000**
OTU17	*Bifidobacterium coryneforme* (99)	7,768	0.189	0.00969	17.4	0.00523	17.2	0.00913	22.5	**0.045**	0.839	0.121
OTU23	Flavobacteriales	2,974	0.183	0.00290	36.0	0.00294	26.3	0.00422	33.1	0.978	0.461	0.545
OTU55	*Gilliamella apicola* (98)	38,619	0.176	0.04133	7.1	0.03045	8.1	0.05264	21.1	**0.017**	0.356	0.078
OTU21	*Dysgonomonas*	3,944	0.173	0.00231	43.2	0.00541	37.8	0.00706	56.9	0.228	0.318	0.791
OTU1	*Lactobacillus apis* (99)	131,977	0.163	0.13239	11.0	0.15581	12.7	0.17471	14.8	0.486	0.162	0.670

**Notes.**

aOTU, number of sequences in a standardized database; the detailed description of the OTUs is given in the raw dataset provided in Table S2.

The heatmap (Fig. S6A) shows the relative abundance of 24 OTUs selected based on a total abundance equal to or greater than 2,000, and the heatmap after LOG2 transformation (Fig. S6B) shows all of the OTUs. The heatmap confirmed the results of the METASTATS analysis; however, there is some variability between samples.

### Population-level analyses of *M. plutonius*

Based on random forest algorithms, *M. plutonius* (OTU4) exhibited the highest mean accuracy for all of the OTUs analyzed using the EFB factor (Table 2). The relative number of sequences was highest in the colonies in the outbreak zone with clinical signs (EFB2), whereas the numbers in the colonies from EFB apiaries without clinical symptoms (EFB1) were greatly decreased, and the lowest number or an absence of sequences was observed in the control apiaries outside the outbreak zone (EFB0). *Melissococcus plutonius* (OTU4) is not visible in the Krona projections (Fig. S4) of the samples without clinical symptoms, i.e., control (EFB0) and asymptomatic samples (EFB1), but it formed 3% of the bacterial community in the samples with clinical symptoms (EFB2). METASTATS analyses (Table 2) indicated that the coded effects of the EFB factors were significant in terms of relative numbers at the *P* < 0.05 level; specifically, EFB2 was different from EFB1 and EFB0, and EFB1 was different from EFB0. This situation is clearly visible in the heatmap (Fig. S6A). However, the comparison of the sequence numbers in the subsample dataset (Fig. S5) revealed that three apiaries from the control colonies outside the EFB zone exhibited 10 to 15 *M. plutonius* (OTU4) sequences, whereas the *M. plutonius*-related sequences were absent in the rest of the control colonies (Table S2).

### Comparison of *M. plutonius* detection via HTS microbiome analysis and conventional PCR

Based on the conventional PCR results (Table S1), *M. plutonius* was detected in all 24 tested samples from EFB2 colonies with clinical symptoms and in none of the six tested samples from EFB1 colonies without clinical symptoms located in the outbreak apiaries, whereas none of the 19 samples from the control colonies was positive for *M. plutonius*.

The HTS approach used to investigate the honeybee microbiome indicated that all of the samples from outbreak sites (coded as EFB2 and EFB1) were positive for *M. plutonius*. However, all six samples from the EFB1 colonies as well as three of the 19 samples from the EFB0 control colonies were found to be positive for *M. plutonius* by HTS.

## Discussion

### The first EFB case arising after 40 years without reported EFB in Czechia was utilized for determination of *M. plutonius* abundance in workers via HTS

Although the samples investigated in this study represent the first verified clinical outbreak of EFB in Czechia after 40 years of absence (Kamler et al., 2016), we stress that the causative agent *M. plutonius* was never absent from Czechia but that EFB may have occurred mainly in enzootic (see further discussion) and non-lethal states. However, this study constitutes the first analysis of EFB epizootiology through investigation of the honeybee worker microbiomes in case and control apiaries using an HTS approach. In an earlier study (Belloy et al., 2007) employing a conventional (hemi-nested) PCR technique, it was not possible to perform quantitative comparisons of *M. plutonius*. The use of the HTS approach enabled us to express the prevalence of *M. plutonius* in workers of EFB-diseased and EFB-asymptomatic colonies. The quantitative advantage of qPCR allowed Roetschi et al. (2008) to identify an increased load of *M. plutonius* in workers collected from brood combs compared with bees near hive entrances. In the present study, according to our HTS analysis, all of the colonies from the two tested apiaries exhibiting clinical EFB symptoms, including EFB-asymptomatic colonies, were positive for *M. plutonius*. Taken together with the fact that HTS enabled the detection of *M. plutonius* in three out of 19 control samples with negative PCR results, we can infer that the absence of the detection of *M. plutonius* by PCR was due to the detection limit. The lack of detection of *M. plutonius* by PCR could influence the lower proportion in the microbiome in EFB0 than in EFB1, although the sequence numbers based on HTS were similar.

The analysis of the worker honeybee microbiomes in the investigated samples revealed a 75-fold higher load of *M. plutonius* in worker bees from colonies exhibiting clinical symptoms compared with EFB-asymptomatic colonies located at EFB-diseased sites. Furthermore, we found that all of the samples from EFB-asymptomatic colonies were positive for *M. plutonius*. These results support the suggestions of previous researchers (Belloy et al., 2007; Roetschi et al., 2008) that it is not only workers from colonies with signs of EFB but also those from EFB-asymptomatic colonies located at EFB-diseased apiaries that exhibit increased abundance of *M. plutonius*. In a previous study, colonies exhibiting clinical symptoms were determined to harbor *M. plutonius* loads greater than 50,000 CFUs per worker bee from brood nests; however, bees from colonies with fewer than 10 visibly diseased larvae had loads that were 100-fold lower than the threshold, or the samples were even negative for *M. plutonius* (Roetschi et al., 2008). It should be considered that the number of *M. plutonius* found in worker bees associated with disease transmission can, like other bee diseases, influence various factors, mainly the hygienic behavior of the colony (Spivak & Reuter, 2001; Waite, Brown & Thompson, 2003).

### Support for the enzootic occurrence of *M. plutonius* and *P. larvae*

The detection of *M. plutonius* in control colonies that had never exhibited EFB symptoms is similar to the results of our recent study of *P. larvae*, which revealed that a HTS analysis supported the enzootic state of this pathogen (Erban et al., 2017a). In the present study, we also detected a small number of sequences corresponding to *P. larvae* in some samples, supporting its enzootic state. The presence of *M. plutonius* in the larvae of certain healthy colonies was previously demonstrated by ELISA (Pinnock & Featherstone, 1984), and this finding was confirmed by hemocytometer and plate counts (Pinnock & Featherstone, 1984). Support for the common incidence of *M. plutonius* in colonies was provided by a previous study conducted in Spain, revealing that the prevalence of *M. plutonius* in both broods and workers was lower than 1%, as demonstrated by PCR (Garrido-Bailon et al., 2013). In contrast, Belloy et al. (2007) and Forsgren et al. (2005) concluded that *M. plutonius* is not ubiquitously distributed. In summary, data obtained from honeybee microbiome analyses can facilitate the evaluation of the occurrence of *M. plutonius* in honeybee colonies in an enzootic state (Pinnock & Featherstone, 1984). Future detailed investigations of the occurrence of *M. plutonius* in honeybee colonies in different areas and countries would provide important information regarding EFB epizootiology.

### Asymptomatic colonies from EFB-diseased sites are at high risk of disease outbreak

In general, the intercolony transmission routes of pathogens in honeybee colonies involve drifting, robbing (horizontal transmission) and swarming (vertical transmission) (Fries & Camazine, 2001). In a variety of honeybee pathogens (viruses, *Nosema* spp., and the parasitic mite *Varroa destructor*), intercolony transmission typically occurs via honeybee workers that do not return to their home colony and instead enter a foreign colony (drifting) (Forfert et al., 2015). Thus, drifting and robbing bees can be considered important factors in EFB transmission between honeybee colonies by worker honeybees, specifically considering the tendency of a decreasing incidence of *M. plutonius* in EFB-asymptomatic colonies distant from EFB apiaries (Belloy et al., 2007; McKee, Goodman & Hornitzky, 2004). We found that the proportion of *M. plutonius* in the microbiome was considerably lower in control colonies than in EFB-asymptomatic colonies in EFB apiaries; therefore, asymptomatic colonies from EFB-diseased sites are at high risk of disease outbreak. Although sanitation measures were applied as part of one study conducted in Switzerland, they were not sufficient to prevent EFB outbreaks the following year in the same apiaries; thus, even after the symptomatic colonies were removed from a diseased site, the danger of an EFB outbreak persisted (Roetschi et al., 2008). It should also be noted that the contamination of honey and beekeeping equipment might contribute to the spread of the disease. Although *M. plutonius* does not form spores, it is able to survive for a relatively long time in the hive, including in deposits of larval feces (Bailey, 1959). The analysis of the EFB outbreak in the Krkonose Mountains National Park, Czechia, in the present study showed that even after EFB-diseased sites were destroyed in 2015, EFB was still observed in 2016 at three surrounding sites approximately 2 km away (Kamler et al., 2016). Therefore, the entire site showing EFB symptoms should be eliminated, and surveillance of adjacent apiaries should also be strongly considered (MA CR, 2003; MA CR, 2013). According to regulations in Czechia (MA CR, 2003; MA CR, 2013), apiaries showing evidence of an EFB outbreak are eliminated by burning, which complicates research on this topic because there is limited time during which to collect samples; nevertheless, we believe that this is an appropriate legislatively regulated step to prevent further disease dissemination.

### Changes in the microbiome structure associated with EFB—proposal of candidates for use as probiotics or agents of secondary infections

The relatively simple honeybee gut harbors a specialized and fairly consistent microbial community of eight to ten species, but a number of environmental bacterial species also participate in the honeybee gut microbiome (Engel et al., 2016; Kwong & Moran, 2016; Moran, 2015). The honeybee microbiome structure can be influenced by various stressors, such as pesticides (Kakumanu et al., 2016), antibiotics (Raymann, Shaffer & Moran, 2017), parasites (Hubert et al., 2016; Hubert et al., 2017) and the pathogenic bacterium *P. larvae* (Erban et al., 2017a). Thus, depending on the occurrence of EFB, interactions between such bacteria and the pathogen are expected. These interactions might be important in disease development, and changes in the proportions of symbiotic bacteria within the microbiome can suggest active participation in disease suppression. Based on the changes in the microbiome structure observed in this study, the symbiotic taxa that were increased in the presence of clinical symptoms of EFB compared with EFB-asymptomatic colonies included *F. fructosus* (OTU5), *L. kunkeei* (OTU9), *G. apicola* (OTUs 3, 16, 55), *F. perrara* (OTU18) and *B. coryneforme* (OTU17). Thus, these taxa can be considered synergic with the clinical symptoms of EFB. The taxa that showed higher proportions in the EFB-asymptomatic colonies than in colonies with clinical symptoms of EFB and the control colonies were *S. alvi* (OTU6), *L. helsingborgensis* (OTU7), *L. kullabergensis* (OTU12) and *L. melliventris* (OTU25). Therefore, it is possible that these bacteria play a protective role in the early stage of infection, and it can be speculated that the abundance of these taxa will decrease after the progression of *M. plutonius* infection. The bacterium associated with infections secondary to *M. plutonius* is *E. faecalis* (OTU10) (Bailey, 1963; Forsgren, 2010; Gaggia et al., 2015; OIE, 2008) because we found it at 4% in the microbiome structure in the presence of clinical symptoms of EFB. It should be noted that this result was affected by the existence of some colonies with clinical symptoms of EFB, as demonstrated in the heatmap. Other taxa such as *C. intestini* (OTU15) and *B. apis* (OTU2) generally exhibited lower abundance in the apiaries with EFB than in the controls; we do not currently have a reliable explanation for this finding other than the possibility that these species can occur at increased levels in virtually healthy colonies compared with EFB-positive apiaries. Taken together, these findings indicate that the observed changes in the honeybee microbiome structure might have implications for EFB disease development and are also useful for targeting bacteria that might be effective as probiotics.

Probiotics have been suggested as a possible tool for improving the resistance of honeybees to entomopathogenic bacteria (Alippi & Reynaldi, 2006; Audisio et al., 2011; Evans & Armstrong, 2006; Killer et al., 2014; Kuzysinova et al., 2016), which provides an opportunity to test the effects of symbionts against *M. plutonius* (Endo & Salminen, 2013; Killer et al., 2014; Wu et al., 2014). Rada et al. (1997) isolated lactobacilli, enterococci, and some bifidobacteria during studies of honeybee gut microbiota; however, these researchers did not perform tests against pathogenic bacteria but only characterized *B. asteroides* using biochemical tests and analyzed the sensitivity of these bacteria to veterinary drugs. A genomic analysis of *B. asteroides* showed a capability for respiratory metabolism and the presence of enzymes for coping with toxic products that arise as a result of oxygen-mediated respiration (Bottacini et al., 2012). Killer et al. (2014) found that some *L. apis* isolates from the honeybee mesoderm inhibit *M. plutonius* and *P. larvae* growth in culture. However, in the present study, a METASTATS analysis showed that the microbiome profiles of *B. asteroides* (OTU33) and *L. apis* (OTU1) were not influenced by EFB factors. Audisio et al. (2011) isolated eight gut *Lactobacillus* and five *Enterococcus* strains from a honeybee worker and showed that *L. johnsonii* isolates inhibit different food-borne pathogens and *P. larvae* and *E. faecalis* strains to produce bacteriocin-like compounds with anti-*Listeria* effects. The findings of the present study, such as the 4% proportion of *E. faecalis* (OTU10) observed in Krona projections of worker bees from colonies with clinical symptoms of EFB, can be connected to increases in *M. plutonius*, but it is necessary to consider the high variability among samples demonstrated in the heatmap. The observed increase in *E. faecalis*, which is taxonomically similar to *M. plutonius*, is in agreement with the fact that this species is a common secondary invader associated with EFB that rarely exceeds the abundance of *M. plutonius* and exerts a supplementary pathogenic effect (Bailey, 1963; Forsgren, 2010; OIE, 2008). Gaggia et al. (2015) identified *E. faecalis* together with *M. plutonius* in diseased honeybee larvae in an atypical case of EFB. However, in contrast to this previous study (Gaggia et al., 2015), we failed to identify *P. dendritiformis* within the honeybee microbiome. Moreover, we failed to identify other suggested secondary invaders within the worker microbiome, such as *A. eurydice*, *B. laterosporus*, and *P. alvei* (Alippi, 1991; Bailey, 1956; Bailey, 1963; Bailey & Ball, 1991; Djordjevic et al., 1998). However, although we did identify *Hafnia alvei* (OTU32) in most samples, these findings did not show statistical significance in relation to EFB, similar to the HTS results reported for AFB (Erban et al., 2017a).

Regarding a recent review by Erler et al. (in press), it is imperative to note that the putative secondary invader *A. eurydice* has been suggested to be confused with *L. kunkeei* or less probable to *F. fructosus*, and the lack of *A. eurydice* detection may support this suggestion. *Lactobacillus kunkeei* is important among the lactic acid bacteria in honeybees, forms biofilms in adult bees (Vasquez et al., 2012) and has been observed to be dominant in honeybee larvae in particular (Endo & Salminen, 2013). It has been indicated that some *L. kunkeei* strains can exhibit antibacterial activity against *M. plutonius* (Endo & Salminen, 2013; Vasquez et al., 2012). Thus, the increase in the proportion of *L. kunkeei* (OTU9) in the microbiome associated with clinical symptoms of EFB in a colony suggests its function in the suppression of *M. plutonius*. Based on a METASTATS analysis, statistically significant trends analogous to the changes in the microbiome structure observed for *L. kunkeei* (OTU9) were found for *F. fructosus* (OTU5), *G. apicola* (OTU3, 16, 55), *F. perrara* (OTU18) and *B. coryneforme* (OTU17). Like *L. kunkeei*, *F. fructosus* is a known component of the larval microbiome (Endo & Salminen, 2013). The colonization of the digestive tract by *L. kunkeei* is suggested to be connected with fructose consumption, and *F. fructosus* and *L. kunkeei* strains have been differentiated by the metabolism of mannitol, sucrose and trehalose (Endo & Salminen, 2013). A study by Olofsson & Vasquez (2008) showed that species of *Lactobacillus* and *Bifidobacterium,* including *B. coryneforme,* showed variations in the stomach depending on the source of nectar and the presence of other bacterial genera within the honeybee, and these researchers were also able to isolate novel lactic acid bacteria (Erler et al., in press; Olofsson & Vasquez, 2008). *Gilliamella apicola* is able to simultaneously utilize glucose, fructose, and mannose and break down potentially toxic carbohydrates, leading to improvements in dietary tolerance and maintenance of the health of its bee host (Zheng et al., 2016). A metatranscriptomic analysis of bee guts showed strong activation of the immune system by *F. perrara* and associations primarily between *G. apicola*, *Lactobacillus* and *Bifidobacterium* and the processing of complex carbohydrates (Emery, Schmidt & Engel, 2017). Thus, the observed increases in *L. kunkeei* (OTU9), *F. fructosus* (OTU5), *G. apicola* (OTU3, 16, 55) and *B. coryneforme* (OTU17) related to EFB colonies with clinical symptoms might be associated with the likely attempts to inhibit *M. plutonius* as a result of dietary composition and/or processing changes in honeybees. The honeybee gut microbiome is essential for saccharide breakdown (Lee et al., 2015). Because there is obvious interplay between the changes in the microbiome and the occurrence of EFB mainly in weak colonies and those with low food reserves in the spring (Bailey, 1960; Poltev, 1950), the impaired balance in the microbiome composition provides a space for pathogens such as *M. plutonius*. Moreover, we speculate that the increase in *F. perrara* in the microbiome structure could act as a driver of the immune system in EFB apiaries. This suggestion is based on the observation that increases in *F. perrara* are correlated with dietary changes and impaired host development (Emery, Schmidt & Engel, 2017).

Despite the observed connection between EFB and honeybee symbionts, we detected no effect of *M. plutonius* on the α-diversity of the bacterial community within honeybee workers. Although ANOSIM showed a significant effect of EFB factors on the bacterial distribution (i.e., EFB2 versus EFB0), RDA did not confirm significant effects of the selected environmental variables. The differences in the results obtained for the comparison of EFB2 and EFB1 levels, when using the Bray–Curtis dissimilarity matrix on the one hand and the Jaccard dissimilarity matrix on the other, may be explained by the different relativization of the original data in the construction of the matrixes. In addition, the Jaccard index gives higher weights to rare species (Chao et al., 2005) whose effects could be studied in more detail in the future. METASTATS analyses of the honeybee worker microbiome revealed differences between core bacteria and non-core bacteria, as classified according to Engel et al. (2016).

It should be noted that based on the results of 16S rRNA analyses, we cannot exclude the possibility that different bacterial strains, including those with weak pathogenic effects (Arai et al., 2012), are likely to be present in the control samples. Strains exhibiting differing pathogenic effects due to geographical and temporal isolation are well documented (Budge et al., 2014; Haynes et al., 2013; Nakamura et al., 2016; Takamatsu et al., 2014).

## Conclusions

This study provided the first analysis of EFB epizootiology using an Illumina amplicon sequencing approach. We were able to show that worker bees from colonies with clinical symptoms exhibited higher loads of *M. plutonius*. Nevertheless, all of the examined EFB-asymptomatic colonies were positive for *M. plutonius*, and positive detection was also observed for control colonies located outside of the EFB zone. Through statistical correlations, we were able show that the occurrence of EFB in colonies influenced the core and environmental bacteria within the worker microbiome structure. The HTS approach permitted the semi-quantitative detection of *M. plutonius* within the honeybee worker microbiome. This study suggests that the metabarcoding analysis of the 16S rRNA gene offers advantages in the detection of bacterial pathogens in bees.

##  Supplemental Information

10.7717/peerj.3816/supp-1 Supplemental Information 1 Supplementary Tables

10.7717/peerj.3816/supp-2 Supplemental Information 2 Supplementary Information

## Additional Information and Declarations

### Data Availability

The following information was supplied regarding data availability: GenBank: SRA project No. SRP093440.
