# Supplementary material for: Bacterial community associated with worker honeybees (Apis mellifera) affected by European foulbrood
Source: PeerJ. 2017 Sep 25;5:e3816. doi: 10.7717/peerj.3816 (PMC5619233; doi:10.7717/peerj.3816)
Supplement: Supplemental Information 2 [file peerj-05-3816-s002.pdf]

Supplementary Information

Journal: PeerJ

Bacterial community associated with worker honeybees (*Apis mellifera*) affected by European foulbrood

TOMAS ERBAN[1,*], ONDREJ LEDVINKA[1,2], MARTIN KAMLER[3], BRONISLAVA HORTOVA[1], MARTA NESVORNA[1], JAN TYL[3], DALIBOR TITERA[3,4], MARTIN MARKOVIC[1], JAN HUBERT[1]

Authors information:

[1]Crop Research Institute, Prague, Czechia

[2]Czech Hydrometeorological Institute, Prague, Czechia

[3]Bee Research Institute at Dol, Libcice nad Vltavou, Czechia

[4]Department of Zoology and Fisheries / Faculty of Agrobiology Food and Natural Resources / Czech University of Life Sciences, Prague, Czechia

*__Corresponding author__:

Tomas Erban

Crop Research Institute

Proteomics and Metabolomics Laboratory

Drnovska 507/73, Prague 6-Ruzyne

CZ-16106

Czechia

E-mail: arachnid@centrum.cz

Figure S1. Rarefaction analyses of *Apis mellifera* samples in control colonies (EFB0). The list of samples is provided in Table S1.

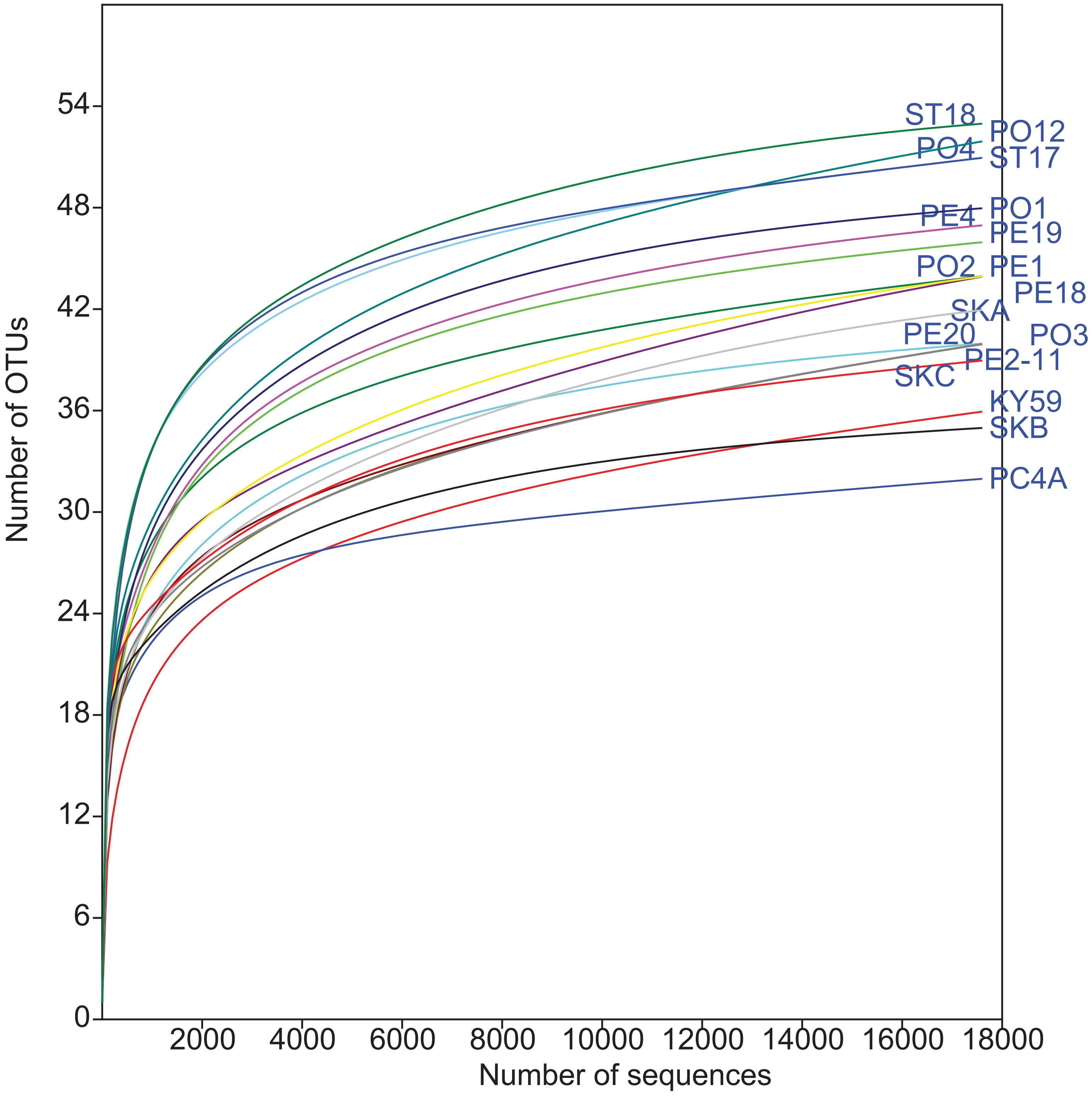

Figure S2. Rarefaction analyses of *Apis mellifera* samples in colonies without symptoms of EFB (EFB1). The list of samples is provided in Table S1.

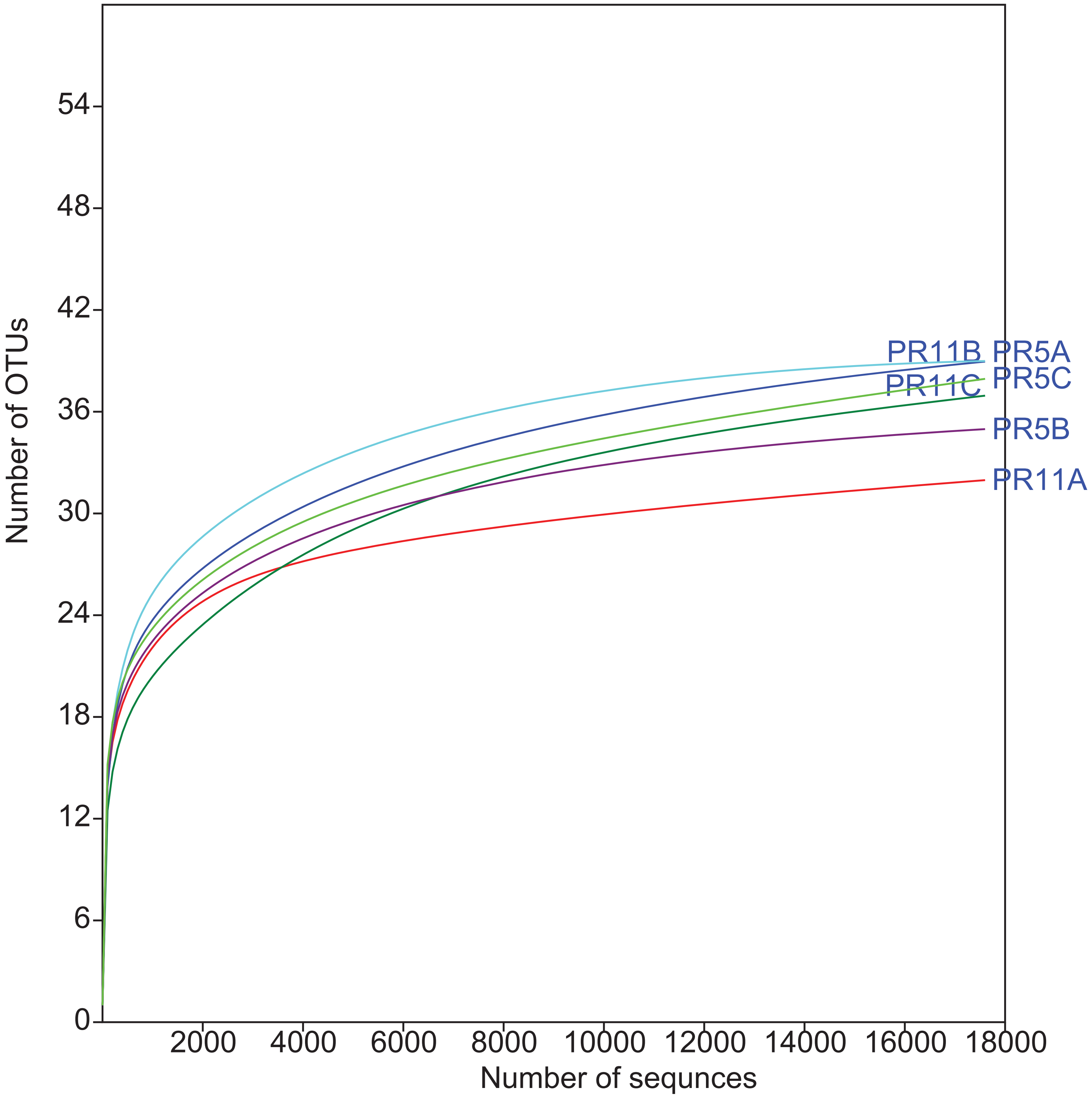

Figure S3. Rarefaction analyses of *Apis mellifera* samples in colonies with clinical symptoms of EFB (EFB2). The list of samples is provided in Table S1.

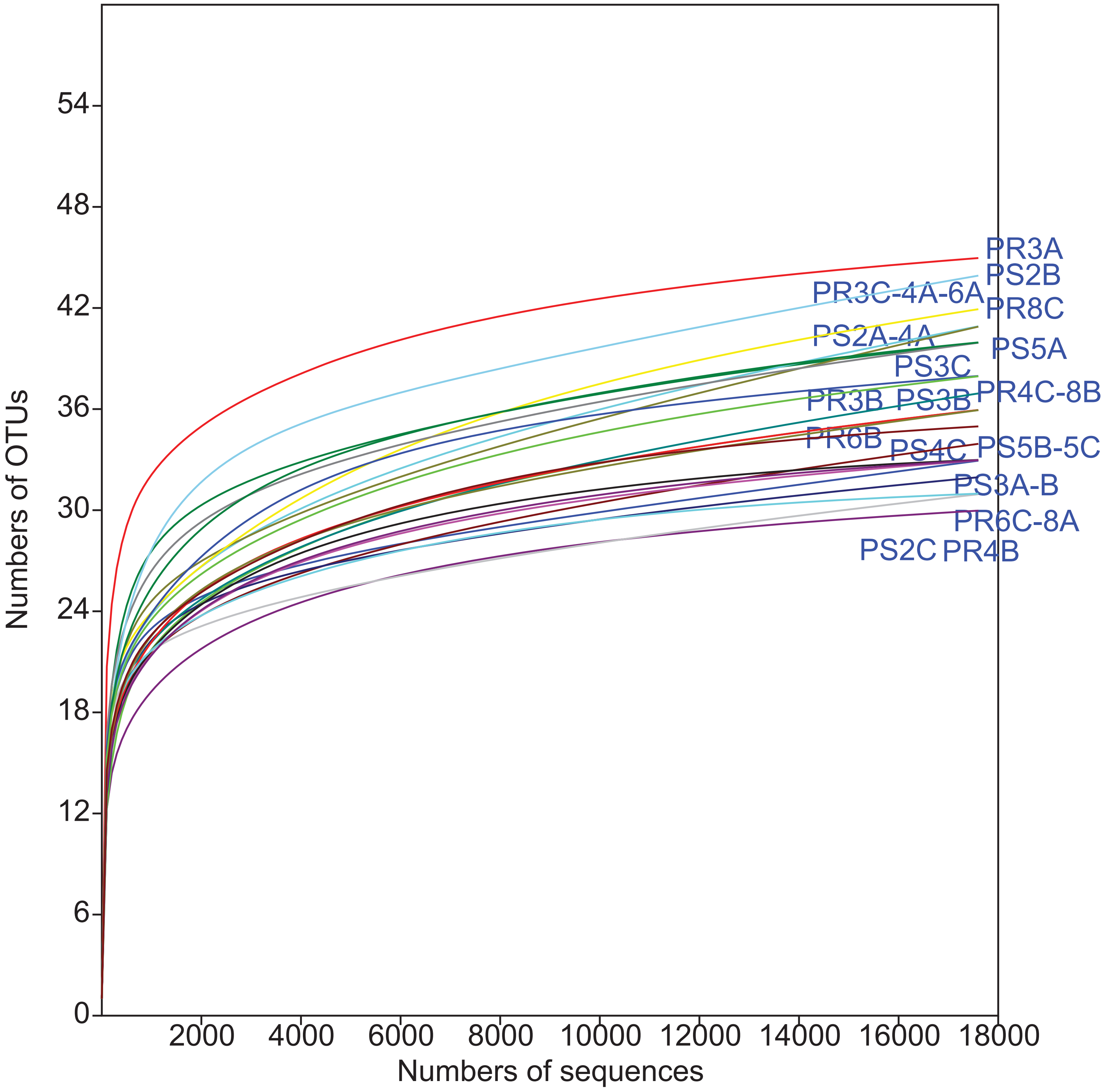

Figure S4. Krona projection of the microbiomes in honeybee worker samples. Mean Krona projections were constructed from subsamples for different situations according to EFB occurrence. Coding for the sample types: EFB0 – control outside the EFB zone without signs of EFB; (ii) EFB1 – bees from an EFB apiary but from colonies without clinical symptoms; and (iii) EFB2 – bees from colonies with clinical symptoms of EFB.

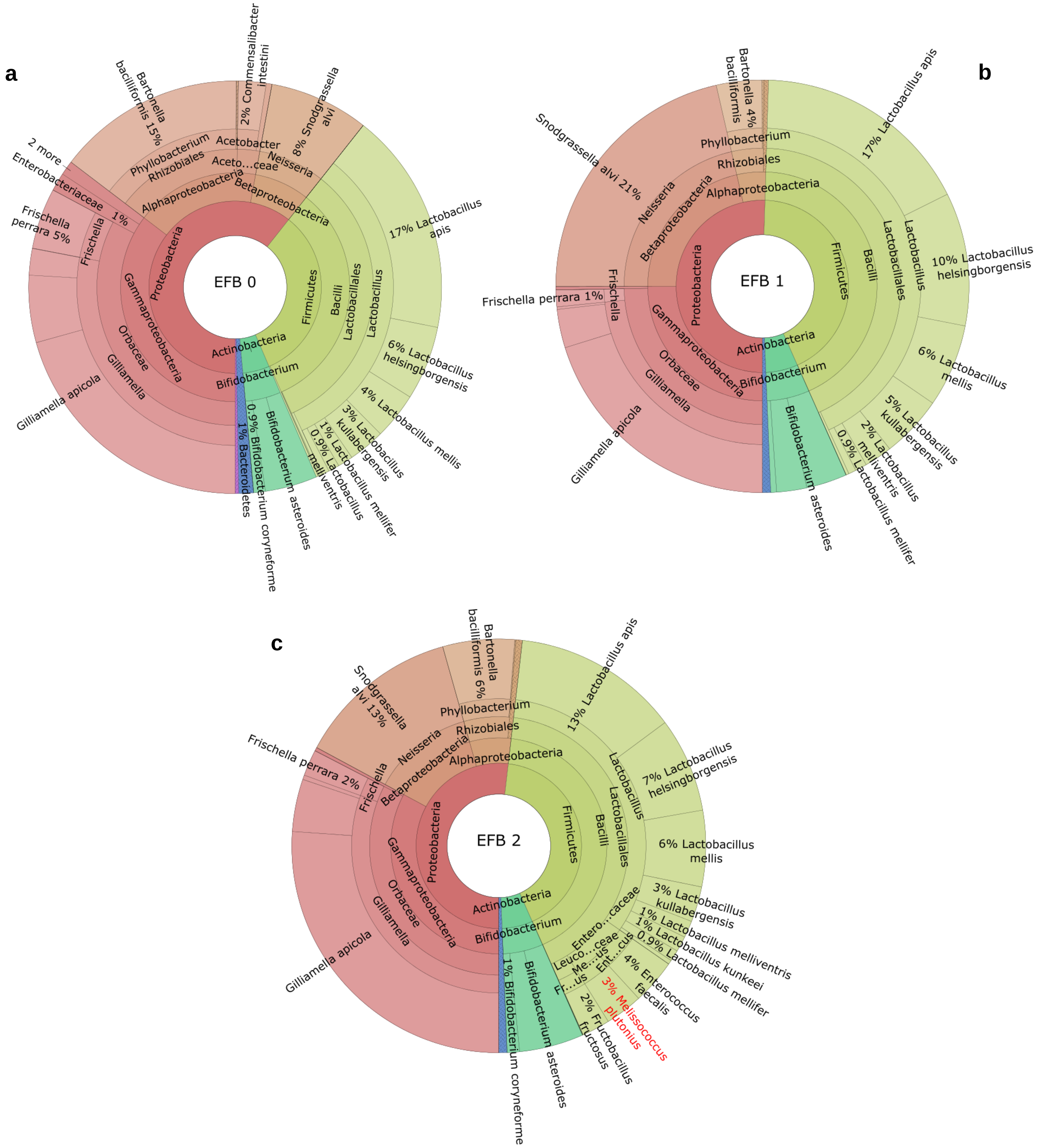

Figure S5. Comparison of the number of *Melissococcus plutonius* sequences in honeybee worker samples.

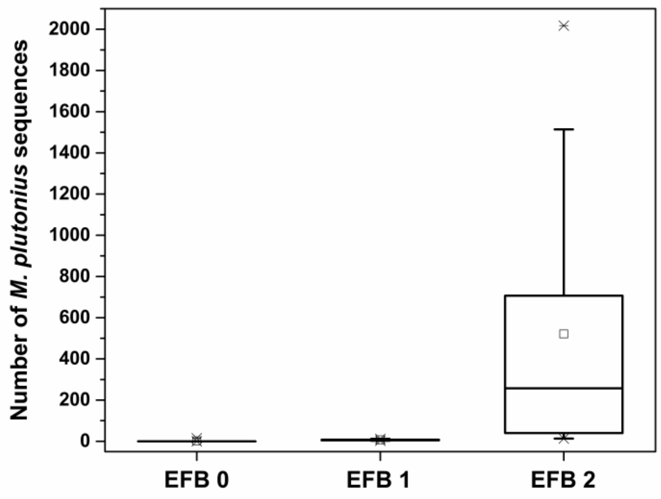

Figure S6. (on next page) Heatmap (**A**) and heatmap in logarithmic scale (**B**) describing the distribution of samples and OTUs in the *Apis mellifera* bacterial community.

Legend: green - EFB0; yellow - EFB1; orange - EFB2.

For the legend of samples, see Table S1 and Table 2.

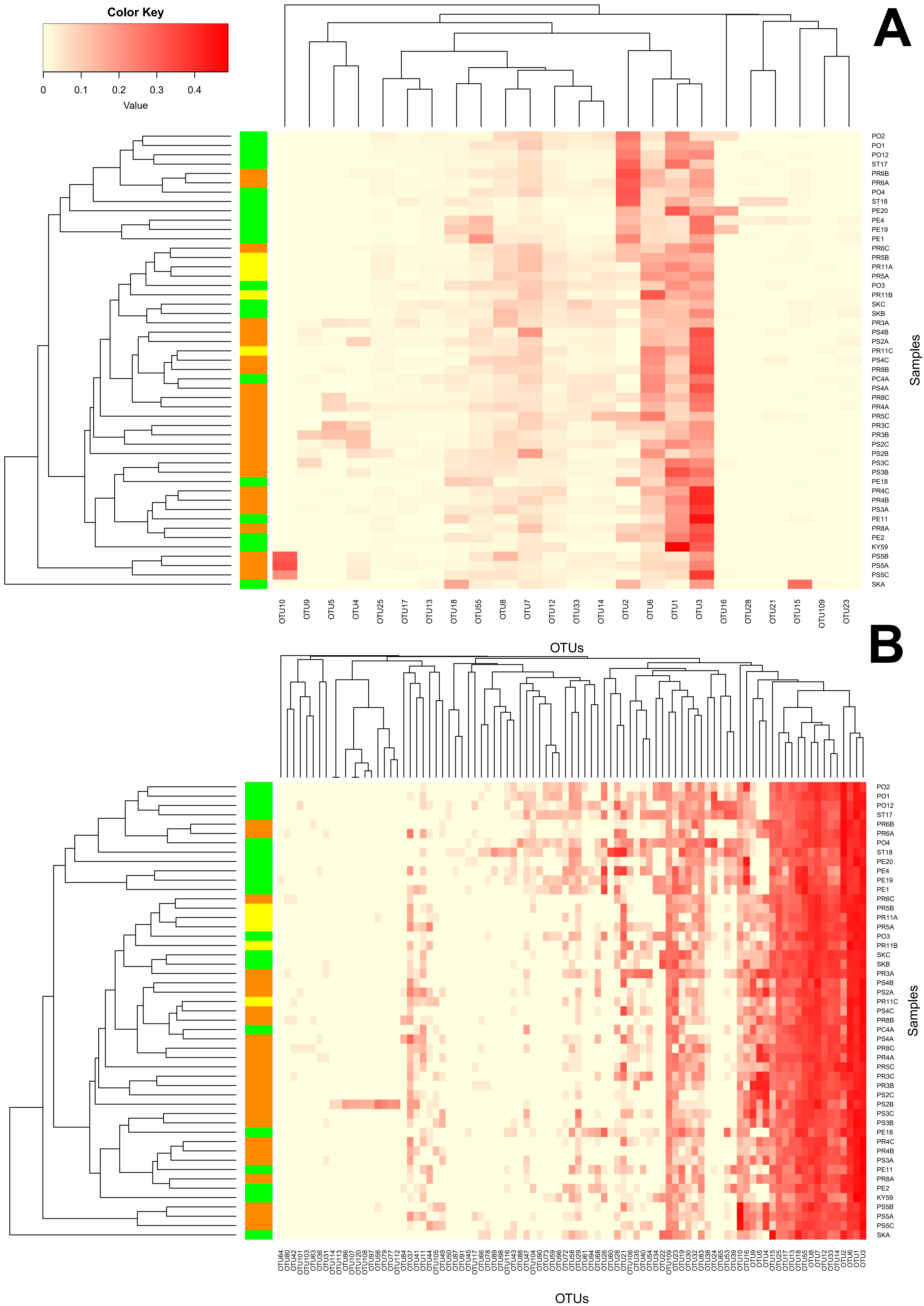
Color Key
0
0.1
0.2
0.3
0.4
Value
A
PO2
PO1
PO12
ST17
PR6B
PR6A
PO4
ST18
PE20
PE4
PE19
PE1
PR6C
PR5B
PR11A
PR5A
PO3
PR11B
SKC
SKB
PR3A
PS4B
PS2A
PR11C
PS4C
PR8B
PC4A
PS4A
PR8C
PR4A
PR5C
PR3C
PR3B
PS2C
PS2B
PS3C
PS3B
PE18
PR4C
PR4B
PS3A
PE11
PR8A
PE2
KY59
PS5B
PS5A
PS5C
SKA
Samples
OTU10
OTU9
OTU5
OTU4
OTU25
OTU17
OTU13
OTU18
OTU55
OTU8
OTU7
OTU12
OTU33
OTU14
OTU2
OTU6
OTU1
OTU3
OTU16
OTU28
OTU21
OTU15
OTU109
OTU23
OTUs
B
Samples
OTUs
